# Electric field promotes dermal fibroblast transdifferentiation through activation of RhoA/ROCK1 pathway

**DOI:** 10.7150/ijms.86215

**Published:** 2023-08-28

**Authors:** Wenping Wang, Wanqi Huang, Jie Liu, Ze Zhang, Ran Ji, Chao Wu, Jiaping Zhang, Xupin Jiang

**Affiliations:** 1 Department of Plastic Surgery, State Key Laboratory of Trauma, Burns and Combined Injury, Southwest Hospital, Army Medical University, Chongqing 400038, China.; 2 Department of Burn and Plastic Surgery, The First Affiliated Hospital of Chongqing Medical University, Chongqing, 400016, China.; 3 Department of Plastic and Maxillofacial Surgery, The Second Affiliated Hospital, Chongqing Medical University, Chongqing, 400010, China.

**Keywords:** Electric field, Myofibroblast, wound healing, chronic wound, RhoA, ROCK

## Abstract

With the increased incidence of age-related and lifestyle-related diseases, chronic wounds are sweeping the world, where recent studies reveal that dysfunction of fibroblast plays an indispensable role. Endogenous electric field (EF) generated by skin wound disrupting an epithelial layer has been used as an alternative clinical treatment in chronic wound by modulating cellular behaviours, including fibroblasts transdifferentiation. Although many molecules and signaling pathways have been reported associated with fibroblasts transdifferentiation, studies investigating how the electric field affects the cellular pathways have been limited. For this purpose, a model of electric field treatment *in vitro* was established, where cells were randomly divided into control and electrified groups. The changes of protein expression and distribution were detected under different conditions, along with Zeiss imaging system observing the response of cells. Results showed that fibroblast transdifferentiation was accompanied by increased expression of a-SMA and extracellular matrix (COL-1 and COL-3) under the EF. Simultaneously, fibroblast transdifferentiation was also consistent with changes of cell arrangement and enhanced motility. Furthermore, we found that electric field activated RhoA signaling pathways activity. Y-27632, a RhoA inhibitor, which was used to treat fibroblasts, resulted in reduced transdifferentiation. The connection between electric field and RhoA signaling pathways is likely to be significant in modulating fibroblast transdifferentiation in acute injury and tissue remodeling, which provides an innovative idea for the molecular mechanism of EF in promoting chronic wound healing.

## Introduction

Wound healing is among the most complex processes in human body, involving the spatial and temporal synchronization of various cell types, which distinctly contribute to three sequential, yet overlapping phases: inflammation, proliferation and remodeling 1. Neutrophils and macrophages are recruited during the inflammatory phase, where they released growth factors and produced cytokines to regulate the subsequent proliferative phase. Then during proliferation phase, fibroblasts and endothelial cells play a pivotal role in granulation tissue, revascularization and mediates wound contraction to bring wound margins together 2. Consequently, the extracellular matrix (ECM) mainly produced by active fibroblasts is remodeled, leading to the tissue repair in the remodeling phase. However, some circumstances, such diabetes and advanced age may lead to faulty healing by deviating the strict regulation, which represents a major and growing cause of morbidity and mortality and burdens the healthcare economy 3. In the United States, non-healing wounds cost the American healthcare system roughly $50 billion annually, scars from trauma and surgical incisions are about $12 billion, and burns are about $7.5 billion 4.

Recent research on chronic wounds has strongly focused on fibroblasts, of which dysfunction can lead to healing delay and even nonhealing wound 5. Fibroblasts are ubiquitously present in the connective tissue of every organ system where they deposit and remodel extracellular matrix (ECM). Current research has demonstrated that fibroblasts are comprised of various lineages, are quite malleable, and are responsive to signals from the epidermis and others within the dermis 1. Myofibroblast, which was first named by Gabbiani et al. in 1971, is a state in which fibroblasts transdifferentiate from a relatively quiet state to an active and overly ECM-producing state when incision occurs as a result of signaling cascades generated by adjacent cells 6. Myofibroblasts are morphologically enlarged and irregular fusiform cells, characterized by actin-based filamentous fibres traversing their entire cytoplasm, such as myosin and α-smooth muscle actin (α-SMA) proteins 7. Myofibroblasts are essential for wound healing and ECM remodeling due to their excessive synthesis of EMC components such as type I and type III fibrillar collagens and contractile smooth muscle. Conversely, increasingly relevant researches indicate that inadequate myofibroblast transdifferentiation, which is consistent with less EMC deposit and delayed wound closure, is to blame for poor healing 5. In light of this, the potential treatment that promoting myofibroblast transdifferentiation provides an alternative approach to chronic wound.

There has been increasing evidence that the biophysical microenvironment (for example, mechanical pressure, liquid shear pressure, and wound hypoxia) may regulate fibroblast transdifferentiation at the injury site 8, 9. When a wound occurs, the role of electric filed caused by the destruction of epithelial barrier cannot be ignored in promoting wound healing. The human skin typically produces between 25 and 40 mV transepithelial potentials (TEPs) due to an asymmetric distribution of ions, such as cation (mainly Na^+^) transported to the basal side and anion (cl^-^) transported to the surface, which is maintained by a resistance barrier formed by tight junctions between epithelium. After wounds, endogenous electric field (EF) are generated naturally resulting from the collapse of TEPs, which point from the edge to the wound center 10. In skin wounds, the endogenous EF intensity remains at about 150-200 mv/mm within 3 days after injury, then gradually decreasing, and completely disappearing after closure 11. There is a certain time-space correlation between changes in EF strength and the fibroblast state. Studies have shown that EF does not compromise cell viability. Within 24 hours of the treatment with EF, the cells exhibit a typical fibroblast morphology with increased directional migration that promotes healing of wounds 12. The intracellular concentration of ECM proteins increases with EF generated by voltage pulses greater than 3V and longer stimulation time than 2 hours 13. Biphasic pulse direct current electric field stimulation results in a directional contraction along fibroblast alignment or the Y-axis of fibroblast populated collagen lattice (FPCL) 14. Previous studies have identified a correlation between electrical stimulation and dermal fibroblast transdifferentiation, but their system did not elaborate on the precise delivery of EF intensities nor focus on specific pathways 15. In this study, we further investigate the precise delivery of electric field intensity and duration, as well as the underlying pathways involved in fibroblast differentiation and wound contraction.

The RhoA GTPase (RhoA) is a widely expressed membrane-associated, guanine nucleotide-binding protein which is essential for the regulation of various cellular functions 16. The members of Rho kinase (ROCK) family, including ROCK1 and ROCK2, are critical effectors of RhoA. The mechanical force activates the RhoA/ROCK2 signaling pathway and then mediates the cell biochemical changes 17. Among these, RhoA is known to have a key role in reorganization of actin cytoskeleton, regulation of cell shape, adhesion, and cellular migration 18. In previously mentioned functions, RhoA is intimately related to cell transdifferentiation 19. Some studies have shown that RhoA promotes the transdifferentiation of cardiac fibroblasts by relying on mineralized corticosteroid receptors to regulate connective tissue growth factor (CTGF) in stomatal cells 19. The reorganization of actin cytoskeleton through the ROS/RhoA-ROCK pathway mediates the toxic effects of fibroblast transdifferentiation and the synthesis of collagen in lung fibrosis 20. RhoA inhibition reduces metabolism and cell migration 20. Various signs indicate that RhoA may be involved in the biological cell behaviors during wound healing, especially cell transdifferentiation. These studies suggest that RhoA pathway may be associated with fibroblast transdifferentiation and wound contraction promoted by EF.

In this study, we employed *in vitro* HSF (human skin fibroblasts) cells to examine the impact of fibroblast transdifferentiation under EF at various time points. The results revealed that EF promoted the skin fibroblasts positive for α-SMA, increased the extracellular matrix containing collagen, and activated RhoA pathway. While RhoA inhibition prevented fibroblast transdifferentiation from being impacted by EF. In summary, we discovered a unique regulatory mechanism behind EF-induced fibroblast transdifferentiation via modulating the RhoA/ROCK1 signaling pathway, thereby uncovering the underlying molecular mechanism targeting fibroblasts under EF in promoting chronic wound healing.

## Methods

### Human Skin Fibroblast Cultures

Normal human skin fibroblasts were obtained from Cell Bank of the Chinese Academy of Sciences in Beijing, China and were maintained in Dulbecco's modified Eagle's medium (C11995500BT, Gibco, Canada) including 10% fetal bovine serum (S-FBS-500, Scitecher, USA) and 1% penicillin streptomycin (GA3502, Genview, Australia). Cell cultures were performed in a 5% CO_2_ atmosphere at 37°C. The medium was changed three times a week. When the culture reached 90% confluence, the cells were separated from the flask with 0.05% trypsin-0.1% ethylenediaminetetraacetic acid (EDTA) solution, washed twice, and then resuspended in DBS supplemented with FBS medium. In each experiment, fibroblasts were used between passages 4 and 5.

### Inhibitor

Where indicated, cells were incubated with pathway inhibitor 10 uM for 30 min 21. The pathway inhibitor: Y-27632 (ab120129, abcam, UK).

### EF stimulation of cell and imaging of single-cell motility

Based on the previous study, a single-phase stabilized direct current power supply (NO: 018101586) was used with EF intensity set at 200mV/mm 22. EF stimulation was given as previously described and fibroblasts were electrified in an chamber made of tissue culturetreated cover slip 23. The EF was used to stimulate cells through two standard silver-silver chloride electrodes in the galvanotaxis chamber containing Steinberg's solution (60 mM NaCl, 0.7 mM KCl, 0.8 mM MgSO4, 0.3 mM CaNO3.4H2O and 1.4 mM Tris base, pH 7.4). That was connected to pools of the excess culture medium on each side of the galvanotaxis chamber by two agr bridges (2% agarin Steinberg's solution). Throughout the experiment, a precision multimeter was placed at both ends of the chamber to regularly monitor the intensity of EF and ensure the stability of EF stimulation on fibroblasts. During the EF stimulation, the Zeiss imaging system (Carl Zeiss Meditec, Jena, Germany) was used for time-lapse imaging, and images were collected every 5 minutes to observe the movement of individual cells. The image was analyzed by image J 1.47v.

### Immunofluorescence staining of α-SMA

The EF-treated and untreated cells were cultured on slips and rinsed twice in pre-warmed (37 °C) PBS. Then these cells were fixed in 4% paraformaldehyde for 20 minutes, permeabilized with 0.3% TritonX-100 for 20 minutes, blocked with goat serum for 1h, and incubated with primary antibodies overnight at 4 °C. For immunofluorescence microscopy, the secondary antibody was coupled to Alexa 488, and the nuclei or whole cell areas were stained with DAPI or cell membrane blue, respectively. Of F-actin labeling fixed cells were incubated with Texas Red phalloidin (1:250, T7471, Thermo Fisher Scientific) for 20 minutes in the dark. The expression of α-SMA, F-actin and DAPI were observed under Leica Confocal Microscope (Leica Microsystems, Wetzlar, Germany). The excitation wavelengths for α-SMA, F-actin, and DAPI were set at 488nm, 680nm, and 405nm respectively, while the corresponding emission wavelengths were 520nm, 720nm, and 450nm. The images were captured with an exposure time of 200ms and a gain of 1.5x. Eventually, the images were processed using Image J.

### Protein extraction and Western blot analysis

Total proteins from cells were extracted using RIPA lysis buffer containing phosphatase and protease inhibitors (Beyotime Biotechnology). The concentration of total protein was detected with a BCA Protein Assay kit (Beyotime Biotechnology). Equal amounts (20μg) of protein were then separated using 4-20% sodium dodecyl sulfate polyacrylamide gel electrophoresis gels. Proteins were then transferred to nitrocellulose membranes. The membranes were blocked with 5% non-fat milk in Tris-buffered saline, and incubated with primary antibodies. Overnight at 4 °C and incubated with the corresponding secondary antibody at room temperature for 1 hour. The molecular imager ChemiDoc TMXRS + imaging system (Bio-Rad) and chemiluminescent reagents detected the signal together. Western blot band intensities were quantified using ImageJ. The using of primary antibodies was as follows: a-SMA (1:1000, ab32575, Abcam, UK), COL-1 (1:1000, ab90395, Abcam, UK), COL-3 (1:1000, 22734-1-AP, Proteintech, USA), RhoA (1:1000, ab86297, Abcam, UK), ROCK1 (1:1000, 21850-1-AP, Proteintech, USA), GAPDH (1:5000, HRP-60004, Proteintech, USA).

### Quantitative analysis of cell migration

We used Image J software to quantify the electrotaxis and motility of cells. The starting point was located at the origin, and the position of nucleus was traced and recorded every 5 minutes. The direction of cell migration is expressed as cosθ, calculated by the cell displacement, which represented the angle between cell moves from the beginning to the end and EF direction indirectly. Ranging from -1 to +1, cosθ objectively quantified the direction of cell migration. To be specific, close to 1 suggested that cell moved towards the cathode while close to -1 towards the anode. Furthermore, close to 0 signified random moves of the cell. Trajectory speed (Tt, Tt/T) was the total length of the track divided by the time, referring to the actual total migration trajectory. Displacement velocity (Td, Td/T) was the distance between the initial and terminal position during observation divided by the time, representing the linear velocity of cell migration.

### Statistical analysis

The statistical analyses were performed using GraphPad Prism version 5.01 (GraphPad Software, San Diego, CA). Data are represented as mean ± standard error of mean (SEM). In order to compare the statistical differences between any pair of data, t-tests and one-way ANOVA test were used to calculate the p-value. P < 0.05 was considered significant.

## Results

### Fibroblasts transdifferentiation was promoted by EF

**Figure 1:**
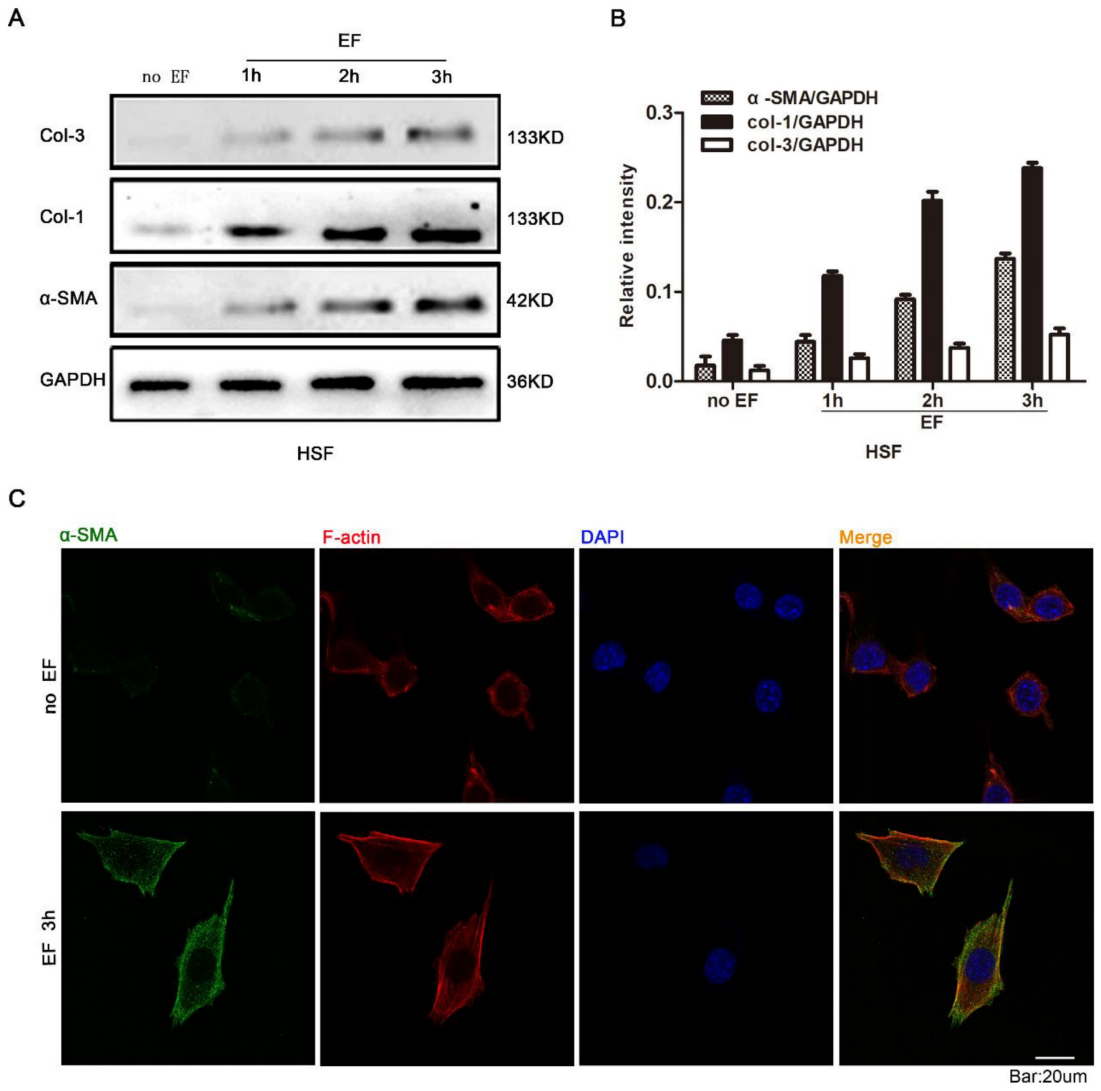
The effect of electric filed on the transdifferentiation of HSFs. The protein expression levels of COL-3, COL-1 and α-SMA in HSFs when treated with no EF, 1h EF, 2h EF and 3h EF were tested by Western blot (A) and the results were quantified by relative intensity (B). The data was shown as the mean±SEM (n=3). The expression and distribution of α-SMA in cells under no EF and electrified for 3h were observed by immunofluorescence (C). Bar=20µm.

In order to investigate whether EF stimulation could cause changes in fibroblast cytology, we observed the response of HSF cells at various time points with EF treatment. Early transdifferentiation into myofibroblasts is characterized by the presence of α-SMA protein and excessive deposit ECM, composed of collagen, elastin, and glycoprotein 24. The HSF cells were electrified for 1h, 2h and 3h, and the expression of α-SMA, COL-1 and COL-3 proteins was analyzed using Western blot (Fig. 1A). The results showed that α-SMA, COL-1 and COL-3 were highly expressed in EF-treated cells but were lower in untreated cells (Fig. 1B). Furthermore, indirect immunofluorescence staining also provided evidence of increased α-SMA protein expression when HSF cells were electrified for 3h (Fig. 1C). In combination with analysis by Western blot, these data strongly indicated that fibroblasts transdifferentiation into myofibroblasts was promoted in response to electrical stimulation.

### Fibroblasts were promoted to migrate and rearrange under EF stimulation

**Figure 2:**
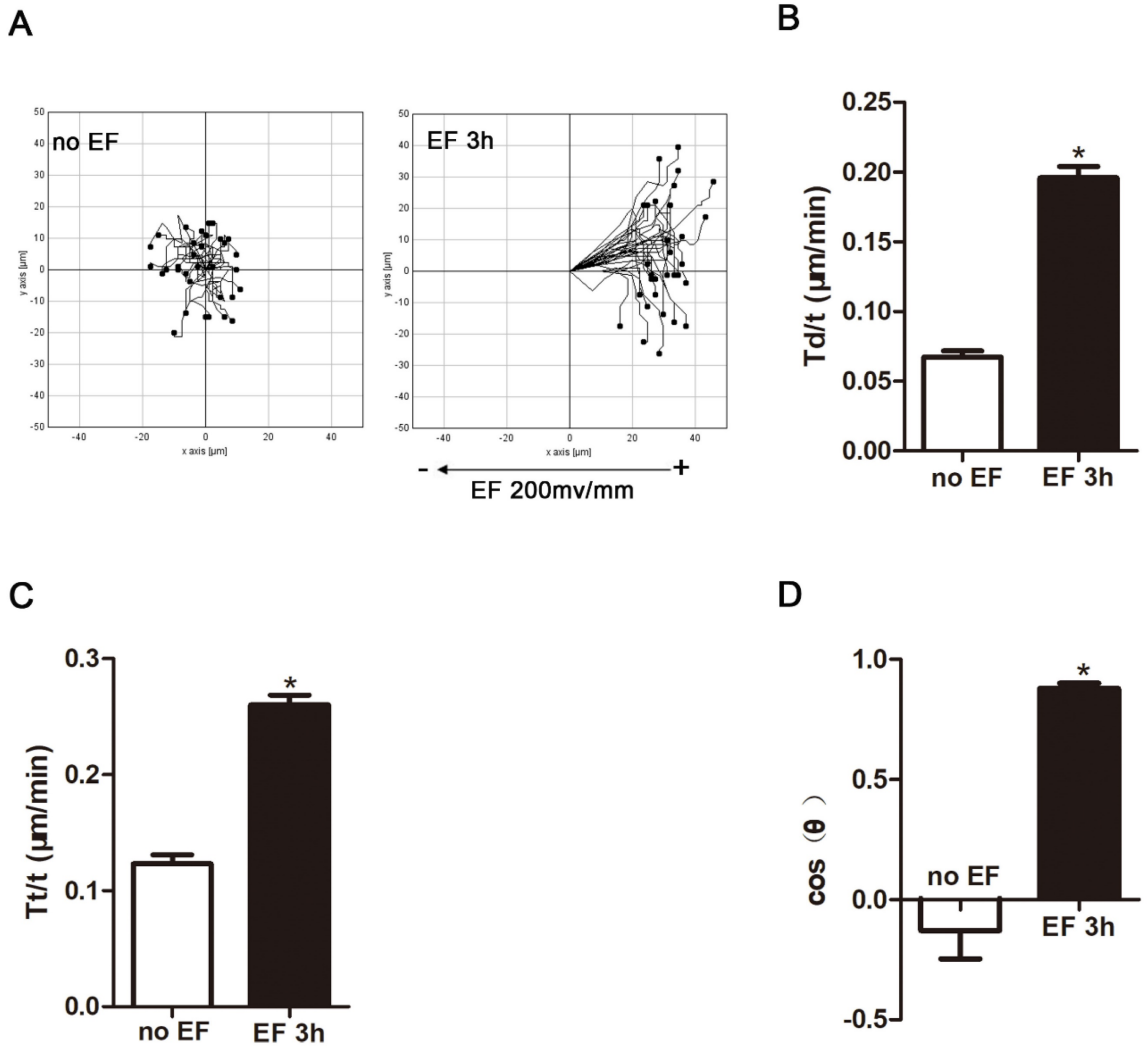
The effect of electric filed on the migration and arrangement of HSFs. When electrified for 3h or not, cell migration was recorded by time-lapse microscopy at 1 frame per 5 minutes and was analyzed by Image J (A). Quantitative analysis of Td/t (μm/min) (B), Tt/t (μm/min) (C) and cos(θ) (D) of HSFs migration. The data was shown as the mean±SEM (n=3).

Many types of cells in the wound respond to applied EF by directional cell migration, a phenomenon termed galvanotaxis or electrotaxis^25^. Fibroblasts are no exception. They migrate to the wound, transdifferentiate into myofibroblast cells, deposit extracellular matrix, and shrink the wound 25. Thus, we used a time-lapse microscope to observe the trajectory of HSF cells that were electrified for 3h and their response to electric field. In the absence of EF, HSF cells under normal conditions still approached their initial position within a limited range. However, after 3 hours of EF treatment, the cells showed a tendency to move toward the positive electrode, and significantly irregular and longitudinal adjustment was observed, indicating that the electric device could effectively modulate the cells (Fig. 2A, Movie S1-2), which is in line with previous findings 25. Cell migration was measured using three typical parameters: trajectory speed (Tt, Tt/T), displacement speed (Td, Td/T), and direction (cosθ) 26. Trajectory speed and the displacement velocity respectively refer to the actual total migration speed and the distance between the initial and terminal position during observation. The cosine value represents the direction of the migration and is determined by the angle between X-axis and the final position over the time. The more cells migrate toward the positive pole, the closer the cosθ is +1, and vice versa 27. The statistical analysis showed that displacement velocity Td/t (μm/min) and trajectory velocity Tt/t (μm/min) increased significantly after EF treatment (Fig. 2B, C), as well as a rise of cosθ to approximately +1 (Fig. 2D). Therefore, combining migration indicators revealed that EF stimulation promoted movement of human skin fibroblasts towards the positive electrodes, accompanied by an increased migration velocity.

### The RhoA/ROCK1 was up-regulated by EF stimulation

**Figure 3:**
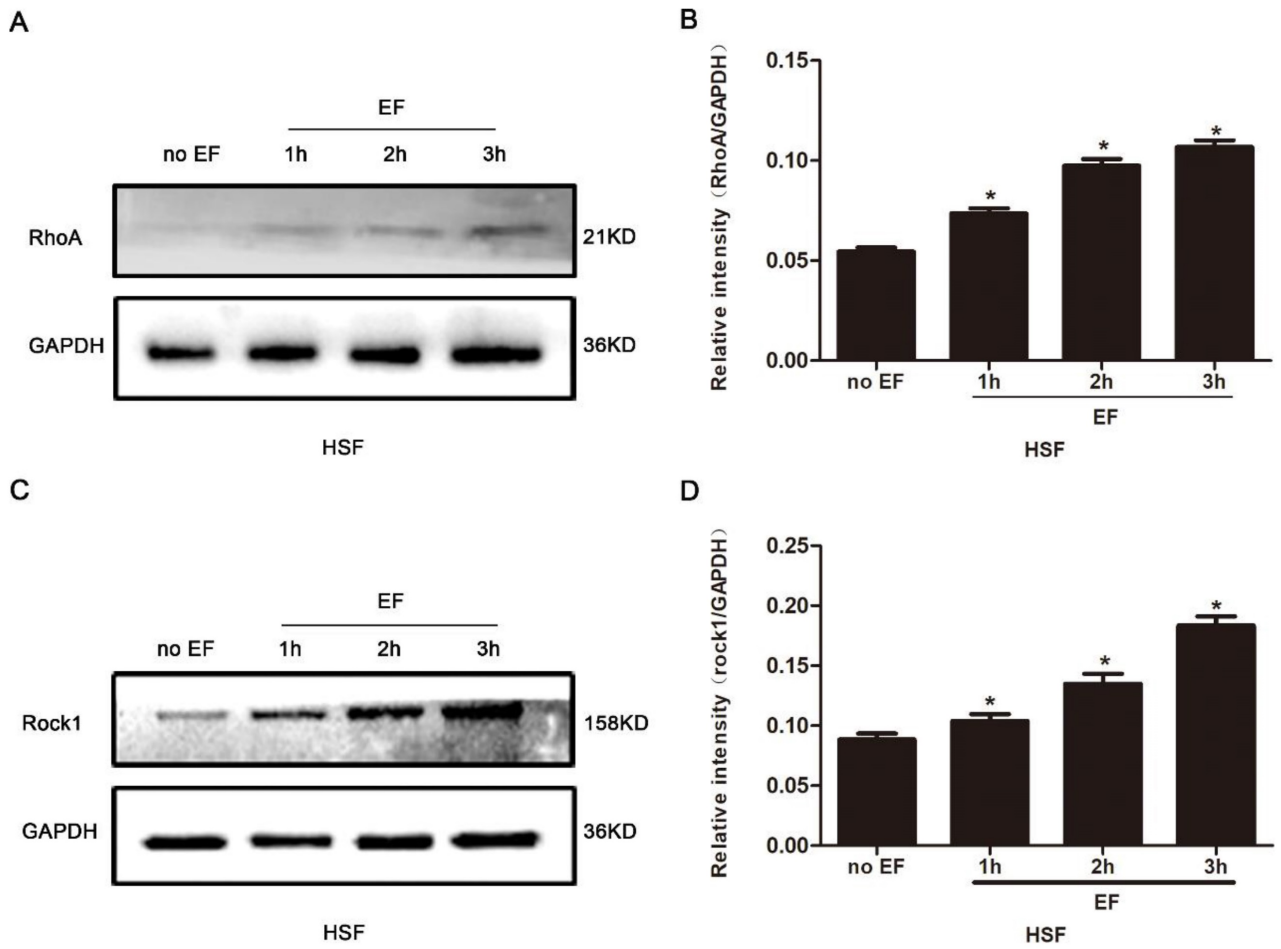
The effect of electric filed on the RhoA/ROCK1 pathway of HSFs. Compared with no EF group, the protein expression of RhoA was enhanced in HSFs when electrified for 1h, 2h and 3h respectively, which was tested by Western blot (A) and the results were quantified by relative intensity (B). The data was shown as the mean±SEM (n=3). *, p<0.05 compared with no EF group. Similarly, Western blot (C) and relative intensity analysis (D) showed increased protein level of ROCK1 when electrified for 1h, 2h and 3h compared with no EF group. The data was shown as the mean±SEM (n=3). *, p<0.05 compared with no EF group.

It is widely reported that the RhoA signal pathway plays a decisive role in cytoskeleton reorganization, transdifferentiation, adhesion and cell migration. Here, we assayed the state of RhoA/ROCK1 signal in the HSF cells treated with EF stimulation for 1h, 2h and 3h, separately. The proteins of RhoA signal pathway were detected by Western blot, and the consistency of the internal reference protein GAPDH across the four groups indicated that the relative expression levels of the loaded protein samples were consistent, allowing for the comparison of RhoA pathway expression levels (Fig. 3A). The level of RhoA in the HSF cells was subordinate in untreated cells, but that increased progressively under EF treatment (Fig. 3B). We also found that ROCK1, an important downstream effector protein of RhoA signaling pathway, showed lower levels in untreated cells, while ROCK1 protein levels in HSF cells increased gradually after EF treatment, which was consistent with the rising of RhoA (Fig. 3C, D). The above analysis indicated that EF had a time-dependent upward regulation of the RhoA/ROCK1 signal.

### RhoA/ROCK1 signaling was involved in EF-induced fibroblast transdifferentiation

**Figure 4:**
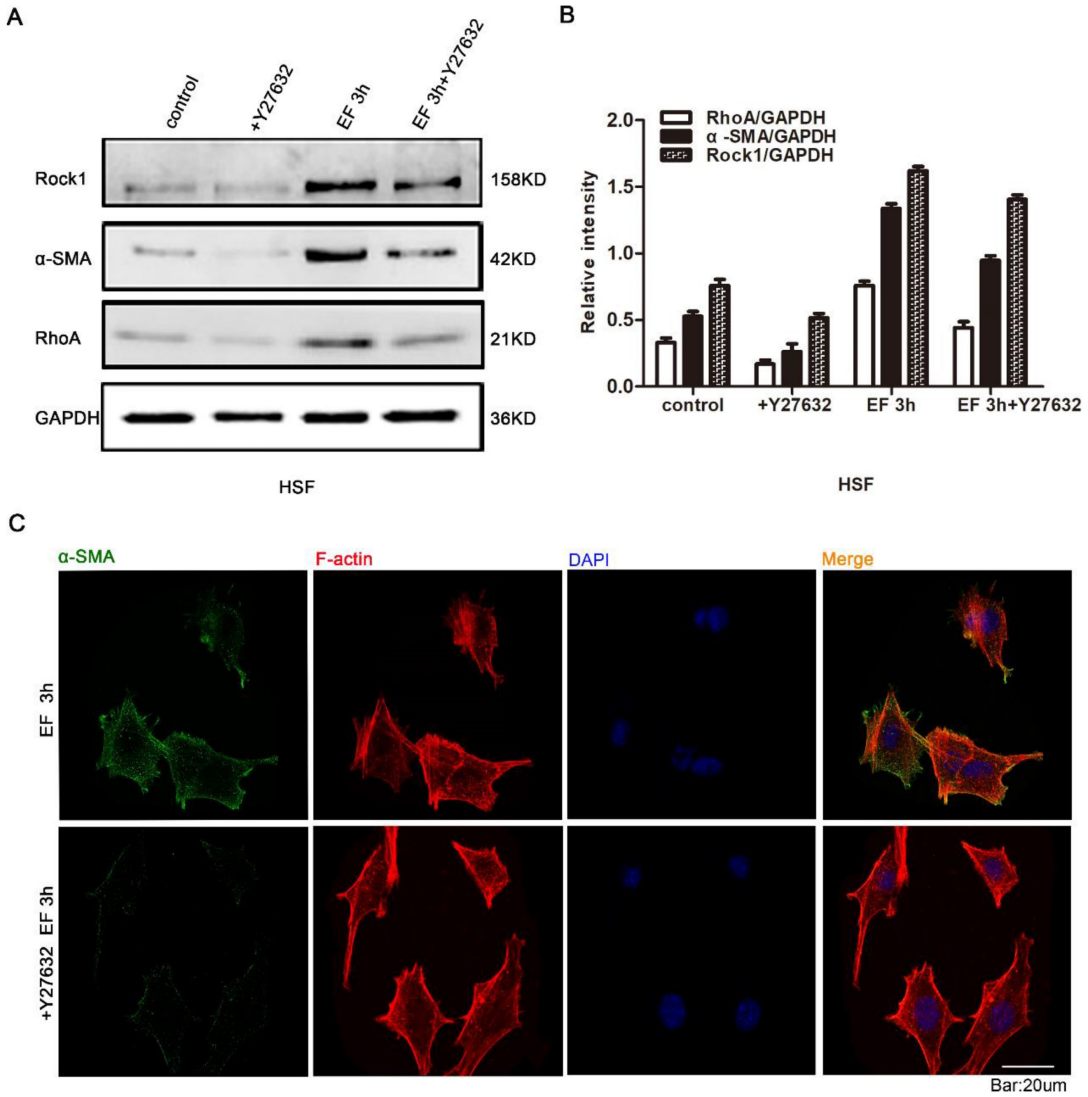
The role of RhoA/ROCK1 pathway in the transdifferentiation of HSFs under electric field. The protein expression levels of RhoA, ROCK1 and α-SMA in HSFs when treated with RhoA inhibitor Y27632 or not and EF or not were tested by Western blot (A) and the results were quantified by relative intensity (B). The data was shown as the mean±SEM (n=3). The expression and distribution of α-SMA in cells when treated with RhoA inhibitor Y27632 or not under electric filed for 3h were observed by immunofluorescence (C). Bar=20µm.

In the stated experiments, we found that EF induced the transdifferentiation of HSF cells and activated the RhoA signal pathway. It is universally recognized that reverse intervention in regulating protein expression is a classic and traditional method for detecting the effect of target proteins. Therefore, in order to study whether the transdifferentiation depends on RhoA signal pathway, Y27632, an inhibitor of RhoA signal pathway, and Western blot were used to detect RhoA signal pathway protein and α-SMA protein. The results showed that RhoA inhibitor Y27632 could prevent the increase of α-SMA and RhoA protein levels in EF-treated cells (Fig. 4A). Quantitative analysis showed that 3 hours after EF treatment, the expression level of RhoA protein in HSF cells increased by 40%. While the expression level of RhoA protein increased by 12% in the cells treated with additional Y27632. Similarly, the α-SMA protein level of fibroblasts in no EF + Y27632 group decreased by 40% compared to no EF group, but EF 3h + Y27632 group represented only 28% reduction compared to the EF 3h group, indicating that EF could reverse the decreased fibroblast transdifferentiation caused by RhoA inhibitor Y27632 (Fig. 4B). In addition, Immunofluorescence staining further supported a decline in the protein level of α-SMA in EF 3h+ Y27632 group, contrasting with cells only electrified for 3h (Fig. 4C). These results suggested that activation of RhoA was related to the transdifferentiation of fibroblasts induced by EF.

## Discussion

Skin fibroblast transdifferentiation and secretion of extracellular matrix are important factors for wound healing, which play a key role in contracting wounds and likely represent potential therapeutic targets for treating chronic wounds 28, 29. Accumulating evidence proves that EF is the one of the most significant factors to regulate fibroblasts behaviours such as migration, secretion and transdifferentiation^13^, ^30^. It has been reported that the RhoA signaling pathway is involved in the transdifferentiation of skin fibroblasts 20, 31. Yet, it is still unclear how EF directs the transdifferentiation of skin fibroblasts. In this study, we demonstrated that EF increased skin fibroblast transdifferentiation via the RhoA pathway, offering an alternative explanation for how an electric field might speed up wound healing.

Following skin damage, a short circuit across the epithelium potential creates a quantifiable endogenous electric field that steadily builds to its peak before gradually fading away until the wound is fully healed 10. Fibroblasts, epithelial cells and endothelium in the damaged skin are actively involved in the angiogenesis, extracellular matrix (ECM) deposition 32, and epithelial reformation 33. EF has been demonstrated to promote epithelial cell migration and accelerate wound healing 34. Recent research demonstrates that the electric field (200 mV/mm) stimulates the expression of the fibroblast proliferation protein, proving that the electric field is not hazardous to skin fibroblasts 15. Further research indicates that fibroblasts exposed to EF may participate in wound closure by activating or transdifferentiating into myofibroblasts, which may be responsible for cell-mediated matrix contraction by contracting actin cellular bone, thereby contributing to tissue repair 14, 15, 35. In our previous study, applying a three-hour electric field could significantly induce the expression of related proteins, so we observed transdifferentiation of skin fibroblasts under a three-hour external electric field with intensity of 200 mV/mm 22. As we hypothesized, skin fibroblasts exposed to EF transdifferentiated into myofibroblasts and produced a large amount of α-SMA and collagens, which were proportional to the length of time. These findings are consistent with the results of an animal study, suggesting that direct current EF is beneficial to collagen synthesis and wound closure 36. But how long it takes for the cells to stop the transdifferentiation of the electric field should be investigated further.

**Figure 5:**
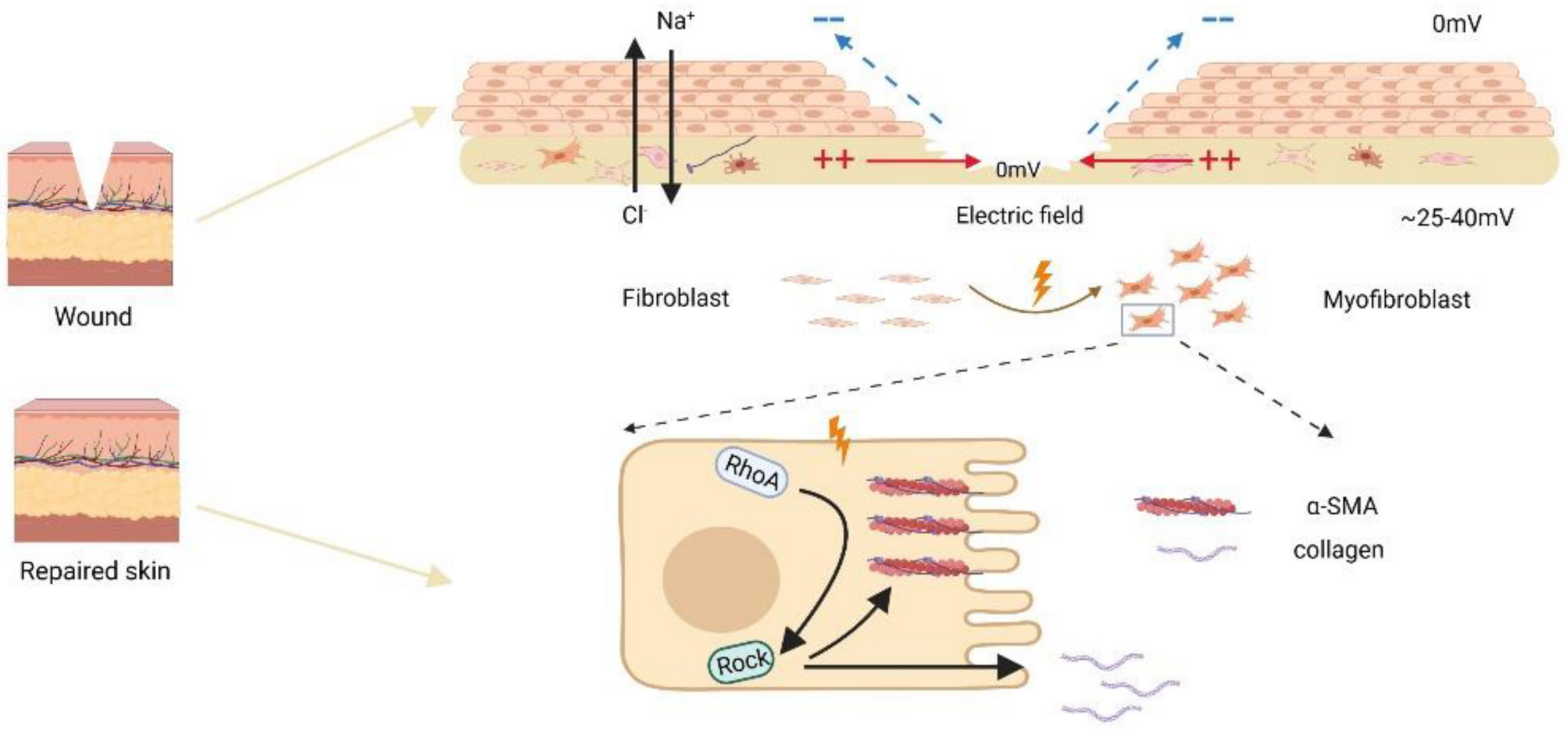
Schematic diagram of electric fields promoting fibroblast transdifferentiation through RhoA/ROCK1 pathway.

Several signaling pathways, including membrane proteins like PI3 kinase/PTEN, cAMP, and Rho small GTPases, have been demonstrated to be involved in the electrotropism of wounded cells in recent investigations 10, 23. Here, we discovered that EF induced transdifferentiation and contraction of skin fibroblasts were related to the activation of RhoA surface pathway. The Rho family of GTPases is widely expressed and composed of 20 members, among which Cdc42, Rac1, and RhoA have been extensively investigated, playing key roles in cellular cytoskeletal remodeling, regulation of cell shape, adhesion, and cell migration 37, 38. Studies have shown that inhibiting RhoA activity can reduce cell migration and delay wound healing, suggesting that RhoA may be involved in the wound repair process 39, 40. Furthermore, RhoA pathway may be involved in the fibroblast transdifferentiation since inhibiting the RhoA pathway can significantly delay pulmonary fibrosis after lung injury 20. In this study, to confirm that role of endogenous EF in promoted skin fibroblast/myofibroblast transdifferentiation and activated RhoA pathway, we used compound Y27632, an inhibitor for RhoA, to treat the skin fibroblast and observed diminished transdifferentiation with RhoA inhibition under EF. Based on our preceding research, we hypothesize that electric field may enhance RhoA activity through upstream molecules and pathways such as transmembrane proteins. In our previous studies, we have demonstrated that EF can regulate the expression of transmembrane protein CD9 to promote cell migration 22. Additionally, other study has shown that transmembrane proteins can regulate cell contraction and actin arrangement through RhoA in human vascular smooth muscle cells 41. Therefore, in our future research, we will focus on the role of EF in regulating upstream molecules of RhoA, such as CD9 and other transmembrane proteins. In a nutshell, we concluded that EF promoted the transdifferentiation of skin fibroblasts through RhoA/ROCK1 signal, which was accompanied by increased secretion of collagens and α-SMA protein levels (Fig. 5).

These results suggested that RhoA pathway played a pivotal role in the regulation of skin fibroblast transdifferentiation under EF. In addition, after the skin incision, the wound microenvironment is complex, including hypoxia and endogenous electric field 42. Along with our findings, it is clear that RhoA signal transduction may play a role in the process of wound healing. Hypoxia and endogenous EF both promote the transdifferentiation of skin fibroblasts through this pathway. The possible effects of hypoxia and EF microenvironment on the transformation of skin fibroblasts need to be further studied. Numerous clinical trials have shown that EF is extremely helpful in the closure of chronic wounds 43. This study reveals the role of EF in skin fibroblast trandifferentiation and its possible mechanism, which will enrich the molecular mechanism of EF in promoting chronic wound healing.

## Supplementary Material

Supplementary movie captions.

Supplementary movie 1.

Supplementary movie 2.
