# Supplementary material for: Electric field promotes dermal fibroblast transdifferentiation through activation of RhoA/ROCK1 pathway
Source: Int J Med Sci. 2023 Aug 28;20(10):1326–35. doi: 10.7150/ijms.86215 (PMC10542021; doi:10.7150/ijms.86215)
Supplement: Supplementary file 1 — Supplementary movie captions. [file ijmsv20p1326s1.pdf]

Supplementary Material

Electric field promotes dermal fibroblast transdifferentiation through activation of the RhoA/ROCK1 pathway

Supporting movie captions:

Supplemental movie 1: HSF cells seldom moved within a small range at the origin without electric fields.

Supplemental movie 2: HSF cells moved toward the positive electrode and became longitudinal under 3 hours of EF treatment.
